# Technical caveats in robot assisted video endoscopic inguinal lymph node dissection - evolution of a modified technique

**DOI:** 10.1590/S1677-5538.IBJU.2019.0298

**Published:** 2020-11-18

**Authors:** Ashwin Sunil Tamhankar, Surya Prakash Ojha, Puneet Ahluwalia, Gagan Gautam

**Affiliations:** 1 Max Institute of Cancer Care Department of Urooncology and Surgical Oncology New Delhi India Department of Urooncology and Surgical Oncology, Max Institute of Cancer Care, New Delhi, India

## Abstract

**Introduction:**

Surgical technique for robotic approach to inguinal lymphadenectomy (R-VEIL) has been adapted and evolved over last few years.

**Materials and Methods:**

We use PDB 1000 balloon® for creation of space below Scarpa's fascia (similar to retroperitoneoscopy). Our approach to lymphadenectomy is “roof first, floor later approach” with separate removal of superficial and deep inguinal lymph node packets, prior to and after opening fascia Lata. Our index case was a 71-year gentleman with T2 disease post partial penectomy with clinically N0 groins who underwent bilateral R-VEIL using the da Vinci Xi® system.

**Results:**

Console times for either side were 98 and 97 minutes, respectively with a total estimated blood loss of 50cc and 2 days of hospitalization. There were no intra or postoperative complications. All 13 lymph nodes removed bilaterally (right side 7, left side 6) were negative for malignancy.

**Conclusion:**

Our technical modification has certain distinct advantages. PDB balloon creates a safe and easy access to create an adequate space. “Roof first, floor later approach” replicates open surgical principles more closely vis a vis an en masse dissection, thereby permitting separate pathological evaluation with implications on adjuvant treatment. Though the simultaneous extraction of superficial and deep nodes without relying of frozen section does not adhere with guidelines for N0 groins, R-VEIL gives opportunity to sample both the packets of nodes thereby increasing diagnostic and therapeutic value simultaneously minimizing false negative rates without adding to excess morbidity of skin necrosis or muscle transposition to cover the vessels.

